# Ferromagnetic ordering in Mn-doped ZnO nanoparticles

**DOI:** 10.1186/1556-276X-9-625

**Published:** 2014-11-22

**Authors:** Xi Luo, Wai-Tung Lee, Guozhong Xing, Nina Bao, Adnan Yonis, Dewei Chu, Jiunn Lee, Jun Ding, Sean Li, Jiabao Yi

**Affiliations:** 1 School of Materials Science and Engineering, University of New South Wales, Kensington, 2 High Street, Sydney, NSW 2052, Australia; 2 Bragg Institute, ANSTO, New Illawarra Road, Lucas Heights NSW 2234, Australia; 3 Department of Materials Science and Engineering, National University of Singapore, 10 Kent Ridge Road, Singapore 119260, Singapore

**Keywords:** Diluted magnetic semiconductor, ZnO, Room-temperature ferromagnetism, Nanoparticles

## Abstract

Zn_1 - *x*_Mn_*x*_O nanoparticles have been synthesized by hydrothermal technique. The doping concentration of Mn can reach up to 9 at% without precipitation or secondary phase, confirmed by electron spin resonance (ESR) and synchrotron X-ray diffraction (XRD). Room-temperature ferromagnetism is observed in the as-prepared nanoparticles. However, the room-temperature ferromagnetism disappears after post-annealing in either argon or air atmosphere, indicating the importance of post-treatment for nanostructured magnetic semiconductors.

## Background

Over the past few decades, diluted magnetic semiconductors (DMSs) have drawn extensive attention due to their potential application in spin-based electronic devices [1-3]. Eu chalcogenide and Eu oxide are pure magnetic semiconductors, which were discovered by Kasuya and Yanase in the 1960s [4]. However, these materials all show a very low Curie temperature (77 K). In recent years, III-V-based DMSs, such as In_1 - *x*_Mn_*x*_As and Ga_1 - *x*_Mn_*x*_As, have been considered as the classical models for magnetic semiconductors, which have demonstrated very promising properties for future spintronics devices. Similar to pure magnetic semiconductors, the Curie temperature of these DMSs is still very low. The highest reported Curie temperature of Ga_1 - *x*_Mn_*x*_As, for example, is around 200 K, which is still much lower than room temperature [5]. In 2000, Dietl et al., using the mean field theory based on the Zener model, predicted that GaN and ZnO are two possible candidates of semiconductor host materials for achieving DMSs with a Curie temperature higher than room temperature [6].

Guided by this prediction, many works based on GaN and ZnO were carried out and indeed room-temperature ferromagnetism has been widely reported [7-12]. ZnO-based DMSs have attracted more attention due to their easy fabrication process and promising properties [13,14]. Mn-doped ZnO is one of the typical examples, which shows room-temperature ferromagnetism [15,16]. Most of the Mn-ZnO thin-film samples were fabricated using the vacuum deposition method, such as sputtering [17-19], pulsed laser deposition (PLD) [20] or molecular beam epitaxy (MBE) [21]. However, the ferromagnetism in Mn-ZnO prepared by the physical method can only be observed when the films were deposited under an oxygen-deficient environment. Clusters or secondary phase is possible to be formed to contribute to the ferromagnetism [22]. Therefore, whether the room-temperature ferromagnetism in these samples is intrinsic or not is still controversial. In fact, Mn-doped ZnO was once reported to exhibit paramagnetic behaviour above 1.83 K [23]. Meanwhile, there is an increasing number of works indicating that the formation of second phases or defects may be the origin of ferromagnetism instead of the inherent property of the system [21,24-28]. In addition, it shows that ferromagnetic ordering is strongly dependent on the parameters/condition of preparation, such as oxygen partial pressure and post-treatment [21]. Compared to the physical deposition approach, chemical synthesis is one of the economical ways for the fabrication of DMSs. It can offer a better controllable material composition and prevent undesirable contamination, as well as avoid a high-temperature vacuum environment that may introduce segregation of metallic impurities [26,29]. The thin-film state of Zn_1 - *x*_Mn_*x*_O has been prepared via direct chemical synthesis by Norberg et al. [29]. The film was formed by spin coating of colloidal Mn^2+^:ZnO quantum dots followed by post-treatment. Room-temperature ferromagnetism was observed in this film [29]. However, compared to the physical preparation methods, the doping concentration of the sample by the chemical method is relatively low. The highest concentration of Mn in the ZnO matrix reported in ref. [29] is as low as 1.3%. It is widely accepted that the concentration of magnetic dopants effectively affects the magnetic property of DMSs [30]. For example, for Ga_1 - *x*_Mn_*x*_As, higher Mn concentration leads to higher Curie temperature [31]. However, increasing the doping concentration of dopants using chemical synthesis is a challenge.

In this work, we have synthesized Zn_1 - *x*_Mn_*x*_O nanoparticles with a modified fabrication process and optimized fabrication parameters to achieve a very high doping concentration of the magnetic dopant without segregation. We also systematically studied the post-treatment effect on the magnetic properties of these synthesized nanoparticles.

## Methods

### Synthesis of Zn_1 - *x*_Mn_*x*_O nanoparticles

Zn_1 - *x*_Mn_*x*_O nanoparticles were fabricated with a method similar to that described previously in ref. [29]. All the chemicals were purchased from Sigma-Aldrich (St. Louis, MO, USA) with 99.9% purity. Mn(OAc)_2_ · 4H_2_O and Zn(OAc)_2_ · 2H_2_O with a variety of ratios were dissolved in dimethyl sulfoxide (DMSO) with a concentration of 0.1 M. Then, the mixture solution was added into 0.55 M tetramethylammonium hydroxide (N(Me)_4_OH · 5H_2_O) dissolved in ethanol for a few seconds with vigorous stirring, which is different from ref. [29]. The reaction was at room temperature which is also different from the temperature of 60°C used in ref. [29]. The fast mixture of the two solutions and the reaction at room temperature are to prevent the growth of the nanoparticles to a large size and to facilitate the doping of Mn in a non-equilibrium process, which may lead to a high doping concentration without precipitation/segregation. Zn_1 - *x*_Mn_*x*_O nanoparticles were then precipitated by adding ethyl acetate, and the precipitates were cleaned with ethanol many times using a centrifuge to make sure that all the excess reactants were removed. The final Zn_1 - *x*_Mn_*x*_O nanoparticles were dried in an oven at 100°C for over 24 h. Post-annealing was performed at 500°C under argon (Ar) or air atmosphere for 1 h. In this work, three samples were mainly prepared. The three samples with Mn doping concentrations of approximately 1.0 at%, 4.9 at% and 9.1 at% were analysed by inductive coupled plasma (ICP). We assign the concentrations as 1 at%, 5 at% and 9 at%, respectively, for convenience.

### Physical property measurement

X-ray powder diffraction data were collected by a PANalytical X'pert multipurpose X-ray diffraction system (PANalytical B.V., Almelo, The Netherlands) using Cu Kα radiation. All X-ray diffraction (XRD) scans were operated under 45 kV and 40 mA in the range of 20° ≤2*θ* ≤85°. Step size and time per step were 0.026° and 99.45 s, respectively. The crystalline size of the samples was determined using transmission electron microscopy (TEM) (Phillips CM200, FEI, Hillsboro, OR, USA). X-ray photoelectron spectroscopy (XPS; Thermo Scientific ESCLAB 250Xi X-ray photoelectron spectrometer, Thermo Fisher Scientific, Waltham, MA, USA) was performed using a monochromatized Al K-alpha X-ray source (*hv*) of 1,486.6 eV with 20-eV pass energy. Magnetic properties of the samples were taken using a superconducting quantum interference device (SQUID; XL-7, Quantum Design, San Diego, CA, USA). X-band (9.5 GHz) electron paramagnetic resonance (EPR) spectra were measured by a Bruker EMX X-band electron spin resonance (ESR) spectrometer (Bruker, Karlsruhe, Germany). The high-resolution synchrotron X-ray powder diffraction experiment was carried out on the PD beamline at the Australian Synchrotron using a wavelength *λ* = 0.6887 Å. Phase identification was carried out using the HighScore Plus program.

## Results and discussion

**Figure 1:**
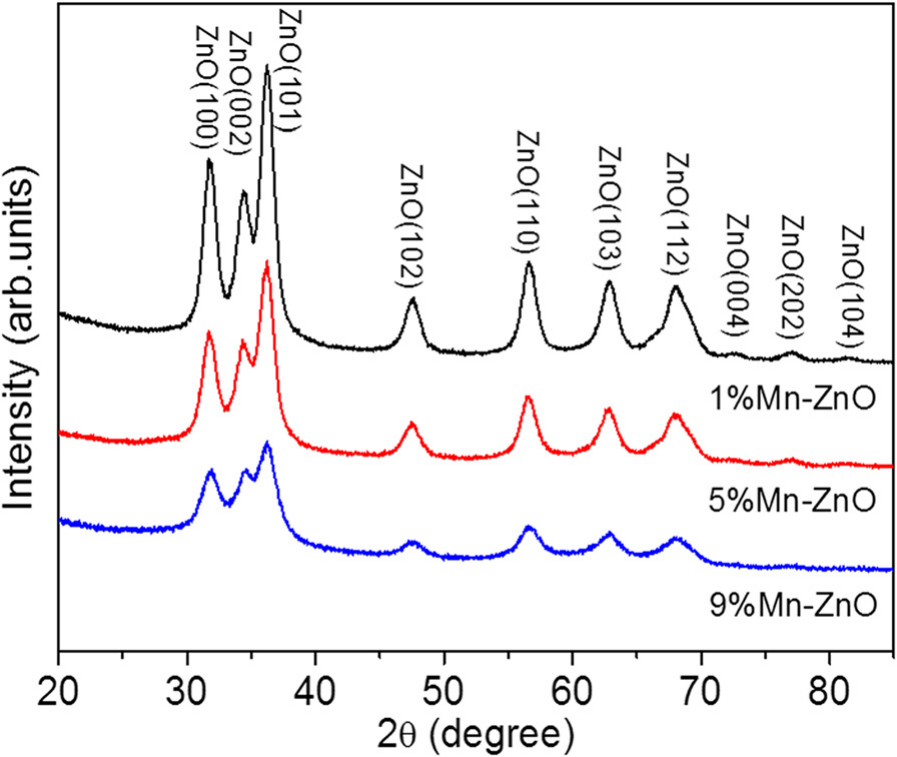
**XRD patterns of Zn**_**1** **-** ***x***_**Mn**_***x***_**O nanoparticles with different Mn concentrations.**

Figure 1 shows the XRD spectra of Zn_1 - *x*_Mn_*x*_O with different doping concentrations. All the samples clearly show a hexagonal wurtzite structure. It is noticed that the crystallinity of the nanostructures decreases as the Mn doping concentration increases. By the refinement using X'Pert HighScore Plus, we calculated the lattice parameters of *a* and *c*. The results indicate that *c* increases with increasing doping concentration. The increase in the lattice parameter may be due to the larger ionic radius of Mn^2+^ (0.83 Å) than that of Zn^2+^ in wurtzite structure (0.74 Å) [32]. However, the lattice parameter *a* has a slight shrink for 9 at% Mn-doped ZnO. It may be because high doping concentration leads to the distortion/disordering of the crystal structure. The mean crystallite size of the sample can be calculated from the Scherrer equation [33].

$$L_{hkl}=\frac{K\lambda }{\beta \cos\left(\theta_{hkl}\right)}$$

*K* stands for the shape factor, which is set to 0.9. *λ* is the wavelength of X-ray radiation of Cu Kα (1.5405 Å). *θ*_*hkl*_ is the most intensive peak. We use the (101) peak in the calculation. *β* presents the full width at half maximum (FWHM) of the peak. We choose peak (101) for the calculation due to its high intensity. The lowest doping sample (1 at% Mn) exhibits the largest crystal size (62.60 Å). With increasing Mn concentration, the crystallite size decreases until it reaches 37.76 Å in diameter for 9 at% Mn-doped ZnO, suggesting that Mn doping may induce distortion of the lattice, leading to disordering.

**Figure 2:**
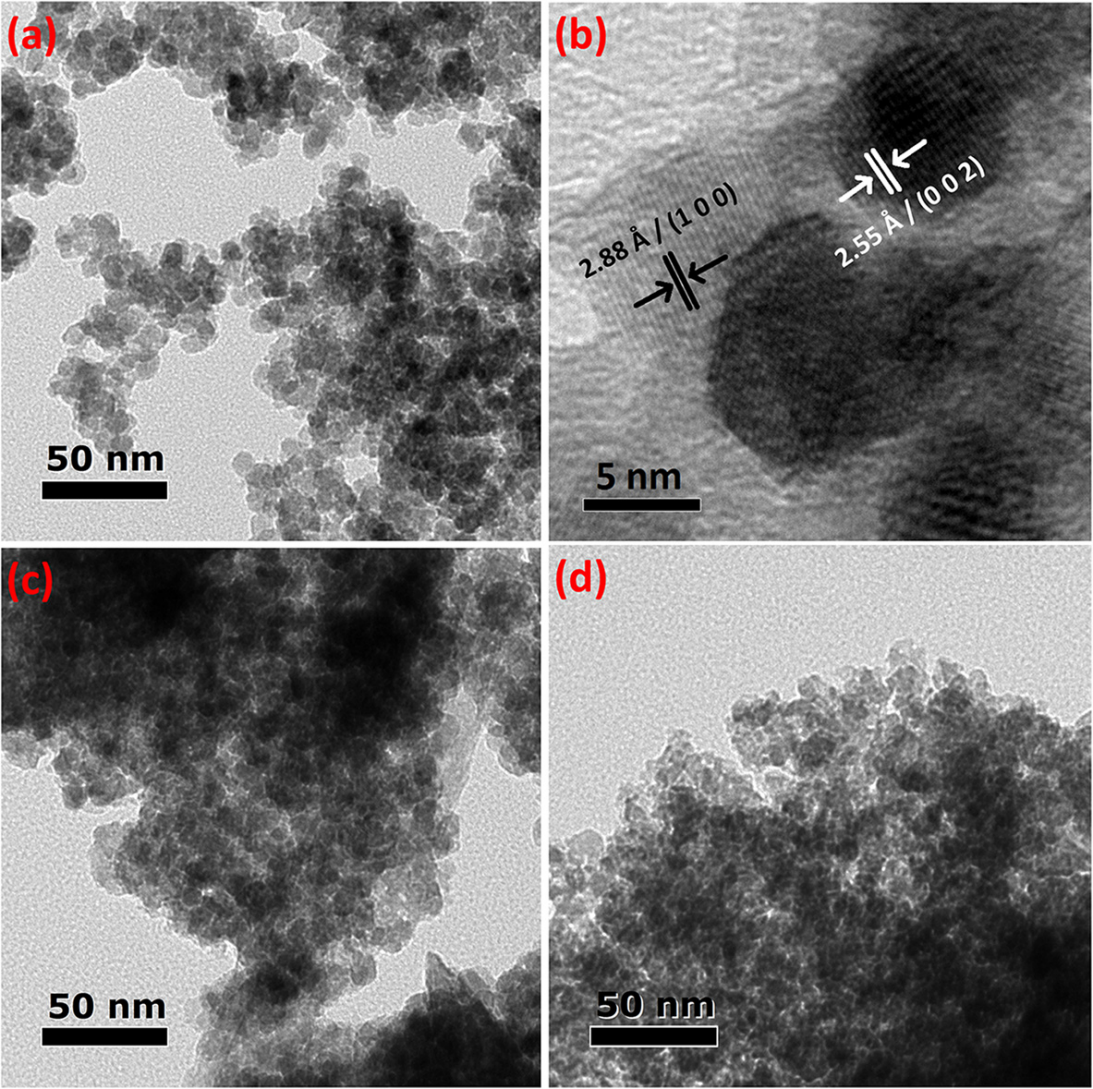
**TEM of Mn-ZnO nanoparticles with various Mn doping concentrations. (a)** 1 at% Mn-doped ZnO. **(b)** High-resolution TEM image of 1 at% Mn-doped ZnO. **(c)** 5 at% Mn-doped ZnO. **(d)** 9 at% Mn-doped ZnO.

TEM is one of the effective techniques for examining the microstructure and identifying the grain size of the nanoparticles. TEM analysis indicates that 1 at% Mn-doped ZnO shows an average grain size of around 7 nm (Figure 2a), which is consistent with that obtained by XRD analysis. The *d*-spacing shown from the high-resolution TEM image is approximately 0.288 nm, which is indexed to the (100) plane of ZnO. When the doping concentration is increased to 5 at%, the crystallite size becomes slightly smaller, estimated to be 6 nm in diameter. When the doping concentration is increased to 9 at%, the image becomes unclear and the estimated grain size becomes smaller. The average grain size is approximately 5 nm in diameter, indicating that increasing doping concentration leads to the refinement of grains.

**Figure 3:**
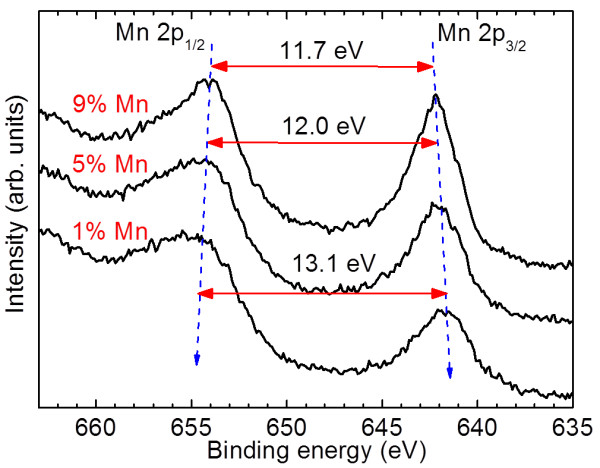
XPS spectra of Mn 2p edge of 1, 5 and 9 at% Mn-doped ZnO nanoparticles.

In order to identify the composition and chemical states of the sample, XPS analysis was carried out. The binding energy of the peaks has been calibrated by taking the C 1s peak (284.7 eV) as a reference. From the spectra as shown in Figure 3, Mn(II), Mn(III) and Mn(IV) may coexist in one multiphase system with a small difference in binding energy. This makes quantitative analysis of XPS of Mn 2p difficult. Therefore, we use the distance of Mn 2p_3/2_ and Mn 2p_1/2_ to identify the oxidation states of Mn instead of splitting the gross peak. The peak of Mn 2p_3/2_ shifts to lower binding energy, while the peak of Mn 2p_1/2_ shifts to higher binding energy when Mn doping concentration increases. Therefore, the spin energy separation between the peaks of Mn 2p_1/2_ and Mn 2p_3/2_ is reduced from 13.1 eV (1 at% Mn-ZnO) to 11.7 eV (9 at% Mn-ZnO), which suggests that the dominant charge state of Mn may shift from Mn(II) to Mn(IV) [34-37].

**Figure 4:**
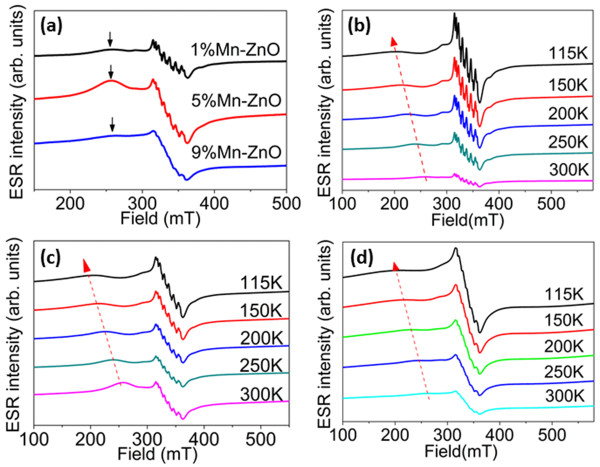
**ESR spectra of Zn**_**1 -** ***x***_**Mn**_***x***_**O with different doping concentrations. (a)** ESR spectra of 1, 5 and 9 at% Mn-doped ZnO nanoparticles taken at 300 K. Temperature dependence of ESR spectra of **(b)** 1 at%, **(c)** 5 at% and **(d)** 9 at% Mn-doped ZnO.

**Figure 5:**
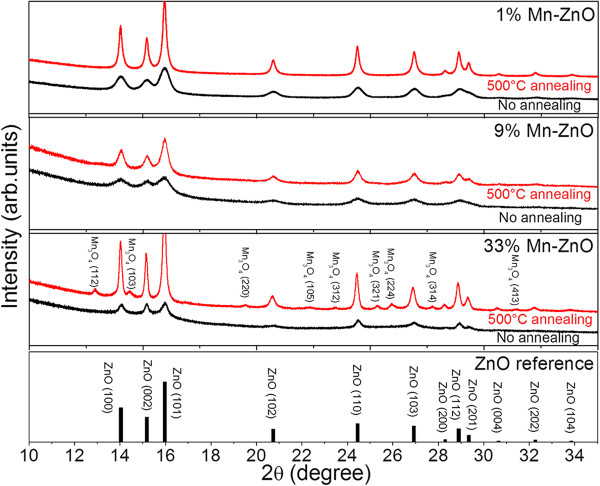
**Synchrotron XRD spectra of 1, 9 and 33 at% Mn-doped ZnO before and after post-annealing.**

In DMSs, the effective doping of dopant plays a vital role in the properties. ESR is one of the most important techniques to determine whether dopants enter the substitutional site. Figure 4 shows the ESR spectra of Zn_1 - *x*_Mn_*x*_O with different doping concentrations. In general, these ESR spectra can be divided into two parts: One is the hyperfine lines in the inclination background in the range of 3,200 and 3,750 G, which indicates the existence of unpaired electrons. The other is a broad hump centred at a magnetic field of 2,500 G that represents spin-orbit coupling [38]. According to Figure 4a, broad humps can be detected in all the samples. The humps at low magnetic field indicates ferromagnetic coupling [39]. The hyperfine transitions in ESR spectra are contributed by the interaction of the Mn^2+^ ion's electron spin and its nuclear spin, ^55^Mn (*I* =5/2) [40]. This indicates that Mn is located inside the nanocrystals and substitutionally replaces the Zn site in the ZnO lattice [41,42]. However, it is noticed that the resolution of the hyperfine lines decreases as the doping concentration increases. It is possible that, in heavily doped samples, some parts of the Mn ions may stay in the interstitial site or on the surface of nanoparticles [41,42]. In addition, the high doping concentration of dopants may lead to the distortion of the lattice and result in a decrease in the resolution of the hyperfine lines. From the XRD analysis, there are no diffraction peaks of secondary phases other than pure ZnO. Hence, the existing oxide should be in amorphous state, given that Mn oxide is indeed on the surface. In order to identify whether there is a Mn oxide phase coated on the surface of the doped ZnO, we performed post-annealing of the synthesized powders at 500°C in Ar atmosphere. Synchrotron XRD is a powerful tool that is used to elucidate the structural change and the existence of possible secondary phases. The spectra are shown in Figure 5. For the 1 at% Mn-doped ZnO, the as-prepared sample and post-annealed sample have similar diffraction patterns except for the increasing intensity of the latter, suggesting an increase in crystallinity and grain size after annealing. No secondary phase was found, indicating the effective doping of the Mn dopant. For the 5 and 9 at% Mn-doped ZnO, similar results of diffraction patterns are observed. No secondary phase is observed either before or after annealing. The only difference is the broadening of diffraction peak in the as-prepared samples, suggesting a smaller grain size. We also employ an extreme case with Mn doping concentration as high as 33 at%. For the as-prepared samples, all the diffraction peaks belong to ZnO, as shown in Figure 5. However, after annealing at 500°C, many small peaks belonging to Mn_3_O_4_ have appeared. For a very high Mn-doped concentration, Mn oxide should be precipitated on the surface of ZnO nanoparticles and the Mn oxide must be in the amorphous state since no diffraction peaks are observed in the XRD analysis. After post-annealing, the amorphous Mn oxide is then crystallized into the Mn_3_O_4_ phase (Figure 5). The results demonstrate that there is no Mn oxide coated on the samples of 9, 5 and 1 at% Mn-doped ZnO since the synchrotron XRD spectra of these samples do not show the Mn_3_O_4_ phase after annealing. As a matter of fact, there are no hyperfine lines in the ESR spectrum for the sample of 33 at% Mn-doped ZnO (not shown here).

**Figure 6:**
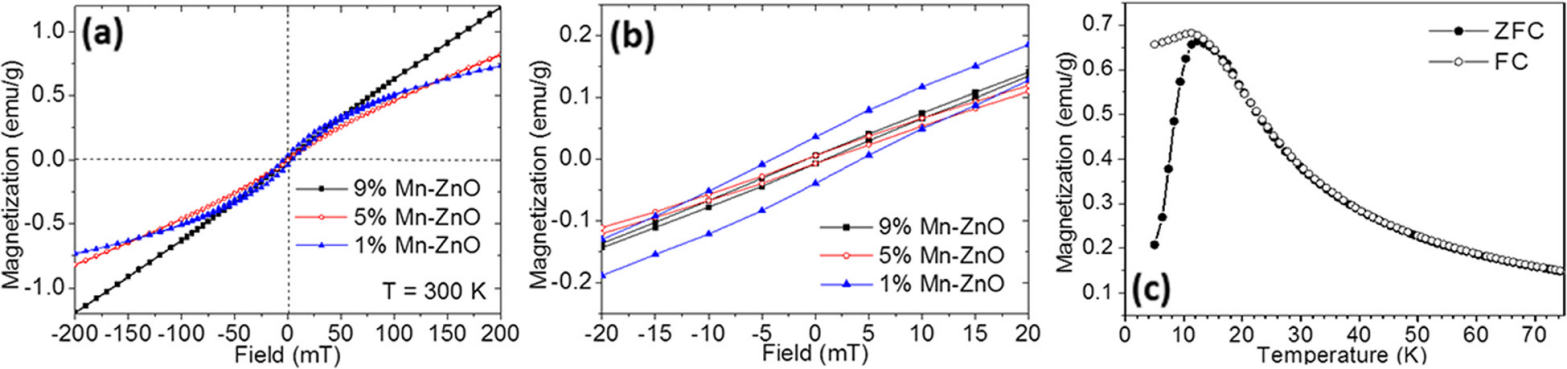
**SQUID measurement of magnetic properties. (a)***M*-*H* curves of 1 at% (blue triangle), 5 at% (red dot) and 9 at% (black square) Mn-doped ZnO nanoparticles taken at 300 K. **(b)** The enlarged area of low magnetic field of (a). **(c)** ZFC and FC plot of 1 at% Mn-doped ZnO nanoparticles measured under an applied field of 20 Oe.

From the ESR spectra as shown in Figure 4b,c,d, it is found that with decreasing temperature, the centre of the large hump shifts to a low magnetic field. This is a typical sign of ferromagnetic ordering in the sample because spin-orbit coupling becomes comparatively stronger when the kinetic energy of the atom is reduced [39,42]. The very weak hump in 9% Mn-ZnO suggests a very weak ferromagnetic ordering in this sample. In order to confirm the results from the ESR analysis, SQUID is used for the measurement of magnetic properties, as shown in Figure 6. The 1 at% Mn-doped ZnO sample shows a very clear hysteresis loop, indicating the significant ferromagnetic ordering. In the 5 at% Mn-doped ZnO sample, both coercivity and remanence decrease significantly compared to those in the 1% Mn-doped ZnO sample (Figure 6a). The *M*-*H* curve of the 9 at% Mn-doped ZnO sample is nearly a straight line. However, for enlarging the low magnetic field area, small coercivity and remanence can still be observed, indicating partial ferromagnetic ordering (Figure 6b). Apparently, when Mn doping concentration is increased, room-temperature ferromagnetism is gradually suppressed and the linear part in the *M*-*H* curves indicates that there is a large amount of paramagnetic signal in Zn_1 - *x*_Mn_*x*_O nanoparticles. This result is also proved by zero field cooling (ZFC) and field cooling (FC) plotting in Figure 6c. ZFC and FC curves of the 1 at% Mn-doped ZnO sample indicate a blocking temperature of 12 K. For temperature above the blocking temperature, two curves seem to be superimposed. This suggests that the dominant phase is the ferromagnetic phase below 12 K, but the paramagnetic phase dominates above 12 K; the SQUID measurement agrees well with ESR results.

**Figure 7:**
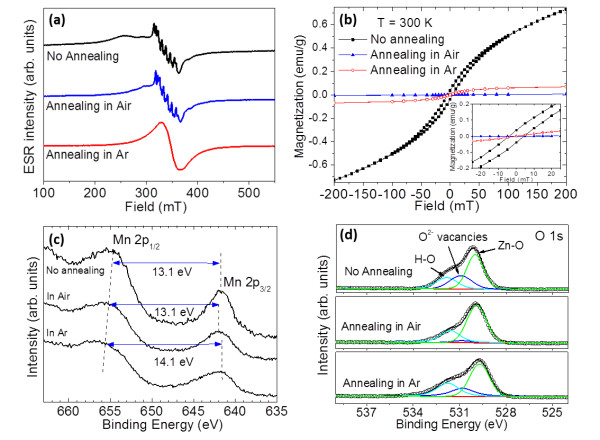
**Results of annealing of 1 at% Mn-doped ZnO under Ar and air atmosphere. (a)** ESR spectra of 1 at% Mn-ZnO without and with annealing in air and Ar atmosphere, respectively. **(b)***M*-*H* curves of 1 at% Mn-doped ZnO with and without annealing. The inset shows the enlarged area of the low magnetic field of (b). **(c)** XPS spectra of Mn 2p of 1 at% Mn-ZnO with and without annealing. **(d)** XPS spectra of O 1s of 1 at% Mn-ZnO with and without annealing.

For the study of ferromagnetism in nanostructured DMSs, post-annealing is of importance. In some cases, ferromagnetism can only be observed after post-annealing [24]. We then performed annealing of 1 at% Mn-doped ZnO under Ar and air atmosphere. The ESR results are shown in Figure 7a. After annealing, the broad humps disappear under either of the atmosphere, suggesting the disappearance of ferromagnetism. The SQUID measurement confirms the non-ferromagnetic behaviour of the sample after annealing, as shown in Figure 7b. According to the inset in Figure 7b, both coercivity and remanence were eliminated after post-annealing, confirming the disappearance of ferromagnetic ordering after annealing. It is worthy to note that the hyperfine lines still exist for the sample annealed in air. It has been claimed that the origin of high-temperature ferromagnetism in the Mn-Zn-O system is an oxygen-vacancy-stabilized metastable phase, Mn_2 - *x*_Zn_*x*_O_3 - *δ*_[43]. After annealing in air, this metastable phase may change from magnetic phase to non-magnetic phase due to the uptake of oxygen [43]. Different from the sample annealed under air atmosphere, the hyperfine lines disappear after the sample is annealed under Ar atmosphere, suggesting that substitutional Mn may precipitate from the Zn site onto the surface of ZnO nanoparticles and form Mn oxide particles. The room-temperature ferromagnetism therefore disappears, which is confirmed again by the SQUID measurement (Figure 7b). The *M*-*H* curve of 1% Mn-doped ZnO after annealing in air is almost linear. Nevertheless, the curve of the same sample after annealing in Ar shows a significant susceptibility at a low magnetic field (-50 to 50 mT) but saturates at a higher magnetic field although its coercivity and remanence are still negligible. This is the so-called superparamagnetism, which may be contributed by nanostructured Mn oxide particles created during post-annealing in Ar.

To deeply understand the role of Mn and O in the magnetic properties of the Mn-Zn-O system, the XPS spectra of the Mn 2p and O 1s core level were measured. As discussed previously, we used the distance of Mn 2p3/2 and Mn 2p1/2 to identify the oxidation states of Mn. Figure 7c indicates that the as-prepared samples and annealed samples in air have Mn^2+^ state, whereas the sample annealed in Ar atmosphere has a large distance between the peaks Mn 2p_3/2_ and Mn 2p_1/2_, indicating a lower valence of Mn. Since the ferromagnetism disappears when annealed under both air and Ar atmosphere, the change of Mn state is not attributed to the disappearance of ferromagnetism. From Figure 7d, the gross peak of O 1s can be divided into three components, which refer to Zn-O (approximately 529.8 eV), O^2-^ vacancies (approximately 530.8 eV) and H-O (approximately 531.5 eV), respectively [41,44-47]. After post-annealing in air, the amount of oxygen vacancy was reduced drastically. The result demonstrates the uptake of oxygen or repairing of oxygen-deficient regions during high-temperature annealing. Either case tends to result in a disruption of the oxygen-vacancy-stabilized metastable phase. It has been reported that the OH bond may also possibly be the origin of ferromagnetism in ZnO-based DMSs [44]. However, in our XPS analysis, the amount of OH bond has a negligible reduction, suggesting that OH bonds may not be the origin of ferromagnetism in this work. Cation vacancy may be one of the origins of ferromagnetism [48-52]. However, in our ESR measurement, Zn vacancy (*g* = 2.013) cannot be observed [53]. It may be overlapped with the hyperfine structure of Mn, whereas after annealing, there is no obvious change in the hyperfine structure, suggesting that Zn vacancy is not the origin of ferromagnetism. Oxygen vacancy may play an important role in ferromagnetism since the air annealing condition leads to the disappearance of oxygen vacancy (Figure 7d). It should be noted that for the sample annealed under Ar atmosphere, ESR analysis has shown that Mn dopants leave the substitutional site and transfer to the surface of ZnO nanoparticles, which may be the main reason for the disappearance of ferromagnetism (Figure 7a).

## Conclusions

We have synthesized Zn_1 - *x*_Mn_*x*_O nanoparticles with different doping concentrations. The Mn dopant can reach up to 9 at% without the formation of secondary phase and precipitates. The as-prepared samples all show room-temperature ferromagnetism. Post-annealing in either air or Ar atmosphere will deteriorate the ferromagnetic ordering, supporting the role of oxygen vacancies in ferromagnetism. In addition, post-annealing easily induces surface oxide precipitation. Hence, delicate control of post-annealing in nanostructured DMSs is of importance for achieving ferromagnetic ordering at room temperature.

## Competing interests

The authors declare that they have no competing interests.

## Authors’ contributions

XL did most of the experiments. NB and JD prepared the samples. AY and DC collected the TEM images. JL and SL measured the *M*-*H* loops. WTL and XL did the synchrotron XRD. JY and XL did the data analysis and drafted the manuscript. GX and the other authors gave revisions for the final manuscript. All authors read and approved the final manuscript.
